# Solute carrier family 1 member 1 in renal interstitial fibrosis: evaluating its influence on glutamate uptake and extracellular ion levels

**DOI:** 10.3389/fmed.2025.1694428

**Published:** 2025-12-17

**Authors:** Chun Yan Lv, Xian Kui Cheng, Sen Fang Luo, Wen Xuan Yang, Geng Lin Liu, Ting Ting Huang, Shi Gao Chen

**Affiliations:** 1 Basic Medical College, Chengdu University, Chengdu, China; 2 Clinical Medical College, Chengdu University, Chengdu, China; 3 Department of Pathology, Shandong Provincial Hospital, Jinan, China; 4 Department of Pathology, The Second Affiliated Hospital of Chengdu Medical University & No. 416 Hospital of Nuclear Industry, Chengdu, China

**Keywords:** renal interstitial fibrosis, SLC1A1 protein, amino acid transport, ion concentration, epithelial-mesenchymal transition

## Abstract

**Goal:**

This research sought to examine the role of solute carrier family 1 member 1 (SLC1A1) in renal interstitial fibrosis (RIF), exploring its relationship with fibrosis extent and its influence on amino acids absorption and ion levels.

**Approach:**

Renal tubulointerstitial damage was assessed using tissue microarray analysis combined with HE staining. Immunohistochemistry was used to identify SLC1A1 expression in fibrotic tissues, and its relationship with renal tubulointerstitial damage was examined. Additionally, an *in vitro* fibrosis model was created using HK-2 cells stimulated by transforming growth factor-β1 (TGF-β1). The levels of SLC1A1 protein were evaluated by immunofluorescence staining, while glutamate uptake capacity and extracellular ion and amino acid levels were monitored.

**Finding:**

The expression of SLC1A1 decreases in cases of renal interstitial fibrosis, with a more pronounced reduction as fibrosis worsens. Following 48 h of exposure to TGF-β1 (15 ng/mL), these alterations became even more apparent. At this 48 h mark, the glutamate uptake in the experimental group was significantly lower compared to the control group, while the levels of extracellular Na^+^ and Cl^−^ showed a notable increase. Additionally, aspartic acid (ASP) levels rose, whereas glutamic acid (Glu) levels fell, and cysteine (Cys) levels remained unchanged.

**Conclusion:**

The downregulation of SLC1A1 during renal interstitial fibrosis is inversely related to the severity of fibrosis and impacts both glutamate uptake and the balance of extracellular ions, indicating its potential as a therapeutic target for enhancing kidney function.

## 1 Introduction

Chronic kidney disease (CKD) is a significant public health issue, affecting approximately 10% of the global population (1). Among patients with stage 3 CKD, 11% will eventually develop end-stage renal disease (ESRD) and require dialysis (2). Furthermore, the treatment of ESRD imposes substantial social and economic burdens. CKD is also a prominent trigger for cardiovascular disease and other complications (3–5). However, the currently available therapies to slow the progression of CKD and prevent related complications are limited. Therefore, studying the pathogenesis of CKD and its treatment is a critical step toward reducing medical costs. Renal interstitial fibrosis is a common pathological process in CKD, primarily caused by the activation of interstitial myofibroblasts and the abnormal deposition of extracellular matrix (2, 6, 7). Activated myofibroblasts are the main matrix-secreting cells in renal fibrosis, and it is now believed that they can arise from the epithelial-mesenchymal transition (EMT) of renal tubular epithelial cells (8, 9). Their differentiation is regulated by factors such as transforming growth factor-β1 (TGF-β1) (10, 11). The injury to renal tubular epithelial cells inevitably leads to alterations in their protein expression (12, 13).

Solute Carrier Family 1 Member 1 (SLC1A1) is a major transporter protein found in renal tubular epithelial cells, encoded by the SLC1A1 gene. The SLC1A1 gene is located at 9p24.2 and spans 97,002 bases. It encodes a multi-channel membrane protein consisting of 524 amino acids, with a molecular weight of 57.1 kDa, featuring eight transmembrane segments and two helical hairpin structures. The primary function of SLC1A1 is to act as a glutamate transporter, clearing glutamate (Glu) from the synaptic cleft, maintaining its concentration balance, and preventing toxic damage caused by excessive neuronal excitation (14, 15). The excitatory amino acid transporter 3 (EAAT3), also known as excitatory amino acid carrier 1 (EAAC1), is encoded by the SLC1A1 gene and is expressed in the kidneys, intestines, and brain. Current research has increasingly focused on its role in the brain. Studies have shown that SLC1A1 is involved in regulating the release and retrieval of neurotransmitters, which affects the transmission of neural signals (14). Mutations (genetic polymorphisms) in this gene have been associated with diseases such as obsessive-compulsive disorder and epilepsy (16–19). The protein encoded by SLC1A1, as a Na + −dependent glutamate transporter, can transport cysteine in tumors, promote the synthesis of glutathione (GSH), inhibit the production of reactive oxygen species (ROS), and function under conditions of ischemia, hypoxia, and oxidative stress, thereby promoting tumor development. Furthermore, SLC1A1 is involved in regulating the R-2-Hydroxyglutarate cancer metabolite (R-2-HG) and the hypoxia-inducible factor 1 alpha (HIF-1α)-SLC1A1 axis, further promoting tumor development (20–23).

The protein encoded by the SLC1A1 gene is a member of the dicarboxylate/amino acid:cation symporter (DAACS) family and plays a pivotal role in the proximal renal tubules by transporting hydrogen ions, sodium and potassium ions, as well as glutamate (24), aspartate (ASP), and cysteine (CYS) across the plasma membrane. It facilitates the simultaneous transport of sodium and chloride ions in the same direction, while transporting potassium ions in the opposite direction, thereby significantly contributing to proximal renal tubular transport (25, 26). This protein is involved in potassium excretion, sodium retention, and amino acid reabsorption in the kidneys, as well as maintaining acid–base balance, which aligns with the kidney’s function of “distinguishing and eliminating turbidity.” Our prior research utilizing bioinformatics has revealed that SLC1A1 undergoes changes during the progression of renal fibrosis and is the sole target of significant traditional Chinese medicine (TCM) formulas aimed at clearing turbidity in proximal renal tubular transport. Studies have demonstrated that the protein encoded by the SLC1A1 gene is critical for kidney function, with its functional abnormalities linked to dicarboxylate aminouria (27, 28), a rare kidney disease characterized by disorders in amino acid metabolism. Additionally, SLC1A1 plays a crucial regulatory role in the occurrence and development of clear cell renal cell carcinoma, with its abnormal expression closely associated with cancer progression (29). Despite being an important amino acid transporter and a protein that regulates acid–base electrolyte balance, the role of SLC1A1 in various kidney diseases has not received adequate attention, and its mechanisms of action remain insufficiently explored. To further investigate the alterations of SLC1A1 in the progression of renal fibrosis and its impact on amino acid and ion concentrations, we conducted the following experiments.

## 2 Materials and methods

### 2.1 Materials, equipment, and reagents

Human renal tubular epithelial cells, designated as HK-2, were sourced from Procell. The renal fibrosis tissue microarrays comprised 11 samples of normal tissue alongside 40 samples from diseased tissues. The apparatus utilized included a CO₂ incubator (DG-250E from Chengdu), a clean bench (Haier HCB-900 V), an automatic biochemical analyzer (Roche Diagnostics), and a fluorescence microscope (Olympus IX73). The reagents employed consisted of a specific HK-2 cell culture medium and a glutamic acid-deficient medium (both from Procell), TGF-β1 (Procell), FITC-glutamate (from Xinweichuang Biotechnology), ELISA kits (ZhiYi), and antibodies targeting SLC1A1, *α*-SMA, E-cadherin, along with DAB detection kits (Sangon Biotech).

### 2.2 Experimental methods

#### 2.2.1 Renal fibrosis tissue microarray analysis

Tissue samples from individuals suffering from obstructive nephropathy were gathered from two hospitals located in Sichuan and Shandong, with sections of the cortical-medullary junction (rich in proximal tubules) sliced into 3–5 mm^3^ cubes and routinely embedded in paraffin. Normal kidney tissues located next to tumor sites (excised during nephrectomy) were utilized as control samples and organized into tissue microarrays. The research received consent from patients and ethical clearance from the institutional review board.

Scoring for renal tubular-interstitial injury (30) and SLC1A1 immunohistochemical analysis was conducted as previously outlined. The damage index was determined by summing scores from eight criteria: vacuolar degeneration of renal tubular epithelial cells, necrosis, dilation, atrophy, presence of red blood cell/protein casts, interstitial edema, infiltration of inflammatory cells, and the extent of fibrosis, all assessed in ten distinct, glomerulus-free cortical areas. The lesions were categorized into four groups based on the index: no injury (less than 4 points), mild injury (4–10 points), moderate injury (10–20 points), and severe injury (20–40 points). For the immunohistochemical evaluation, ten non-overlapping images at 200 × magnification per sample were processed with Image-Pro Plus software to measure the average optical density (AOD) of SLC1A1 staining.

#### 2.2.2 Cell culture experiments

HK-2 cells were grown in a specific medium at 37 °C with 5% CO₂ for2 days, selecting cells in the logarithmic growth phase for the experiments. The cells were treated with TGF-β1 at concentrations of 5, 10, and 15 ng/mL for periods of 12 and 48 h (31). To assess SLC1A1 protein levels, immunofluorescence staining was combined with image analysis. The model for fibrosis was established using the 15 ng/mL TGF-β1 treatment over 48 h, categorizing the cells into experimental (treated with TGF-β1) and control groups. Following a 48 h incubation (32, 33), immunofluorescence was used to evaluate the expression of E-cadherin, *α*-SMA, and SLC1A1. ELISA kits were used to analyze the concentrations of amino acids (GLU, ASP, and CYS) and ions (Na+, Cl-, and K+) in the culture media. For the glutamate uptake assays, cells were rinsed with PBS and then incubated in a medium lacking glutamic acid that contained FITC-glutamate at varying concentrations (0, 25, 50, 100, and 200 μM), with uptake monitored via fluorescence microscopy at 24 and 48 h.

### 2.3 Statistical analysis

The results are expressed as mean values along with their standard deviations. To compare groups, *t*-tests or chi-square tests were utilized. Pearson product–moment correlation coefficient was used to analyze the correlation between SLC1A1 and various parameters of tissue chips, as well as the concentration of amino acids and ions in the culture medium. The analysis and visualization were conducted using Graphpad Prism version 10.1.2. A *p*-value of less than 0.05 was deemed statistically significant.

## 3 Results

### 3.1 Patient demographics and tissue injury characteristics

**Figure 1:**
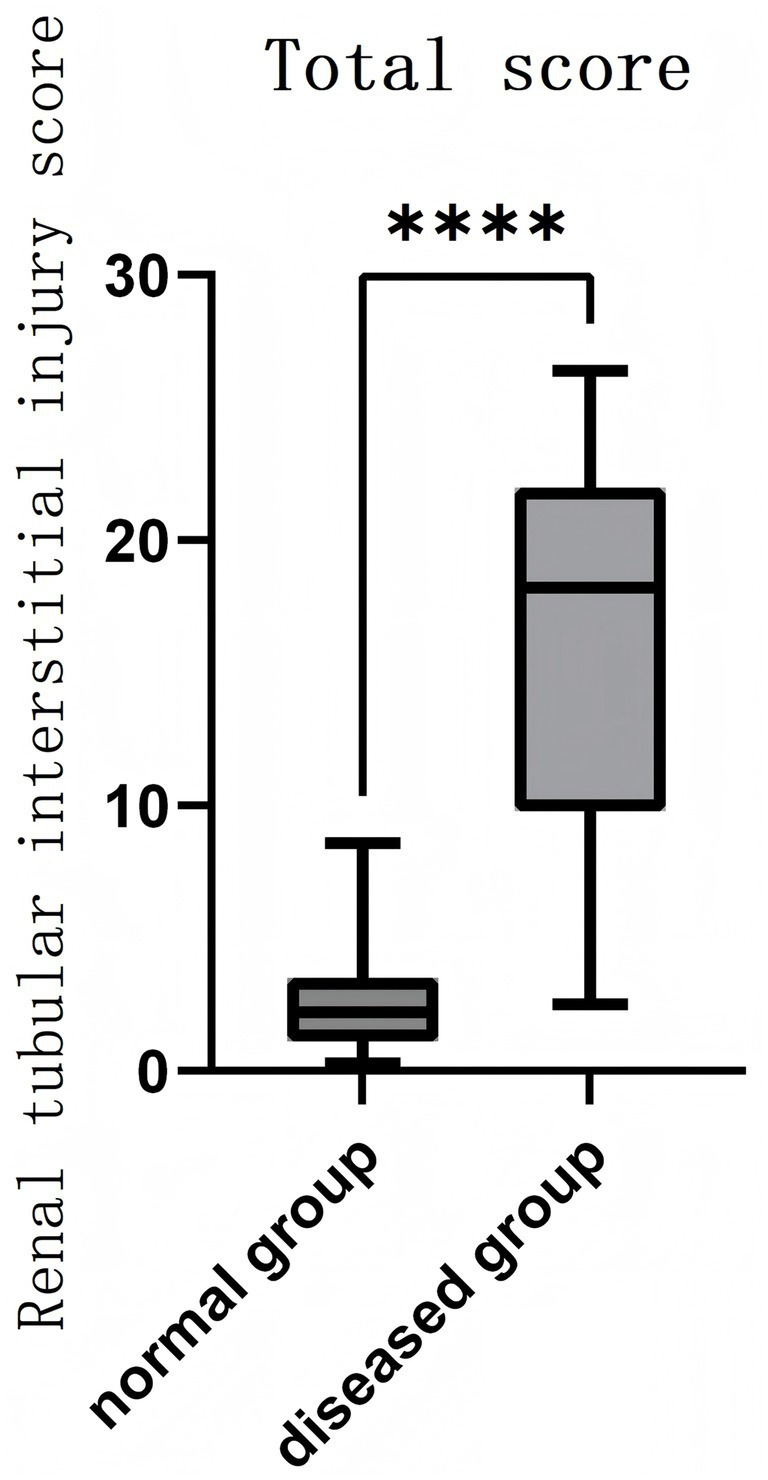
Analysis of renal tubular-interstitial injury scores in diseased and normal tissues. ****p < 0.0001.

A study was conducted involving 51 tissue specimens collected, comprising 11 from healthy individuals and 40 from those with RIF. The analysis revealed no notable differences in gender distribution (healthy: 7 males and 4 females; RIF: 19 males and 21 females) or age (healthy: 56.7 ± 14.6 years; diseased: 55.2 ± 16.0 years) between the two groups (*p* > 0.05). However, the index for renal tubular-interstitial damage was markedly elevated in the RIF cohort (16.7 ± 6.6, range 8.3–26.4) compared to the healthy group (2.1 ± 1.3, range 0.3–3.9) (*p* < 0.05, see Figure 1). Additionally, all eight examined pathological parameters showed significant differences between the groups (*p* < 0.05, refer to Table 1).

**Table 1:** Demographic and pathological characteristics of renal fibrosis patients and normal control group.

Stem		Normal group	Diseased group	Χ^2^/t-Value	*p*-value
Sex	Male	7 (63.64%)	19 (47.50%)	1.183e-031	>0.9999
	Female	4 (36.36%)	21 (52.50%)		
Age		56.7 ± 14.6	55.2 ± 16.0	0.2802	0.7805
Renal tubular interstitial injury index		2.13 ± 1.27	16.72 ± 6.61	7.236	<0.0001
Vacuolar degeneration of renal tubular epithelial cells		0.29 ± 0.33	1.22 ± 0.72	4.126	0.0001
Necrosis of renal tubular epithelial cells		0.11 ± 0.14	2.38 ± 1.26	5.933	<0.0001
Tubular dilation		0.38 ± 0.41	1.63 ± 0.89	4.486	<0.0001
Tubular atrophy		0.24 ± 0.40	1.97 ± 1.25	4.521	<0.0001
Red blood cell casts and/or protein casts		0.14 ± 0.25	1.39 ± 0.88	4.635	<0.0001
Interstitial edema		0.29 ± 0.21	2.22 ± 0.96	6.572	<0.0001
Interstitial cell infiltration		0.47 ± 0.30	3.08 ± 1.22	6.978	<0.0001
Degree of interstitial fibrosis		0.21 ± 0.21	2.99 ± 1.41	6.491	<0.0001

### 3.2 SLC1A1 expression in renal tissues

**Figure 2:**
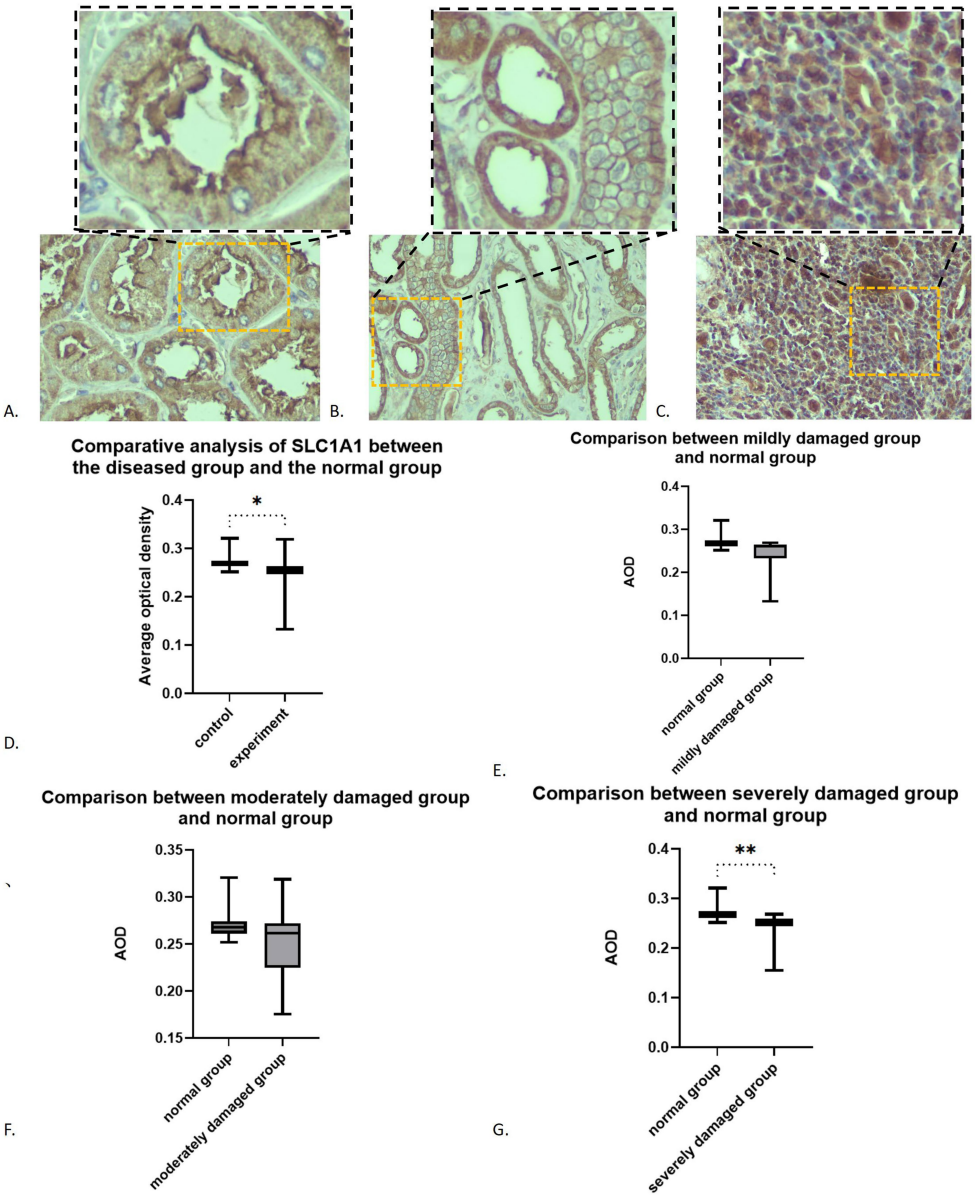
Expression of SLC1A1 in renal fibrosis tissues: **(A)** SLC1A1 in proximal tubule (×200): the image in the upper right corner box is a partial ×400 magnification; **(B)** SLC1A1 in distal tubule (×200): the image in the upper right corner box is a partial ×400 magnification; **(C)** SLC1A in lymphocytes (×200): the image in the upper right corner box is a partial ×400 magnification; **(D)** Overall comparison; **(E)** Comparison between mildly damaged group and normal group; **(F)** Comparison between moderately damaged group and normal group; **(G)** Comparison between severely damaged group and normal group. *p < 0.05; **p < 0.01.

**Figure 3:**
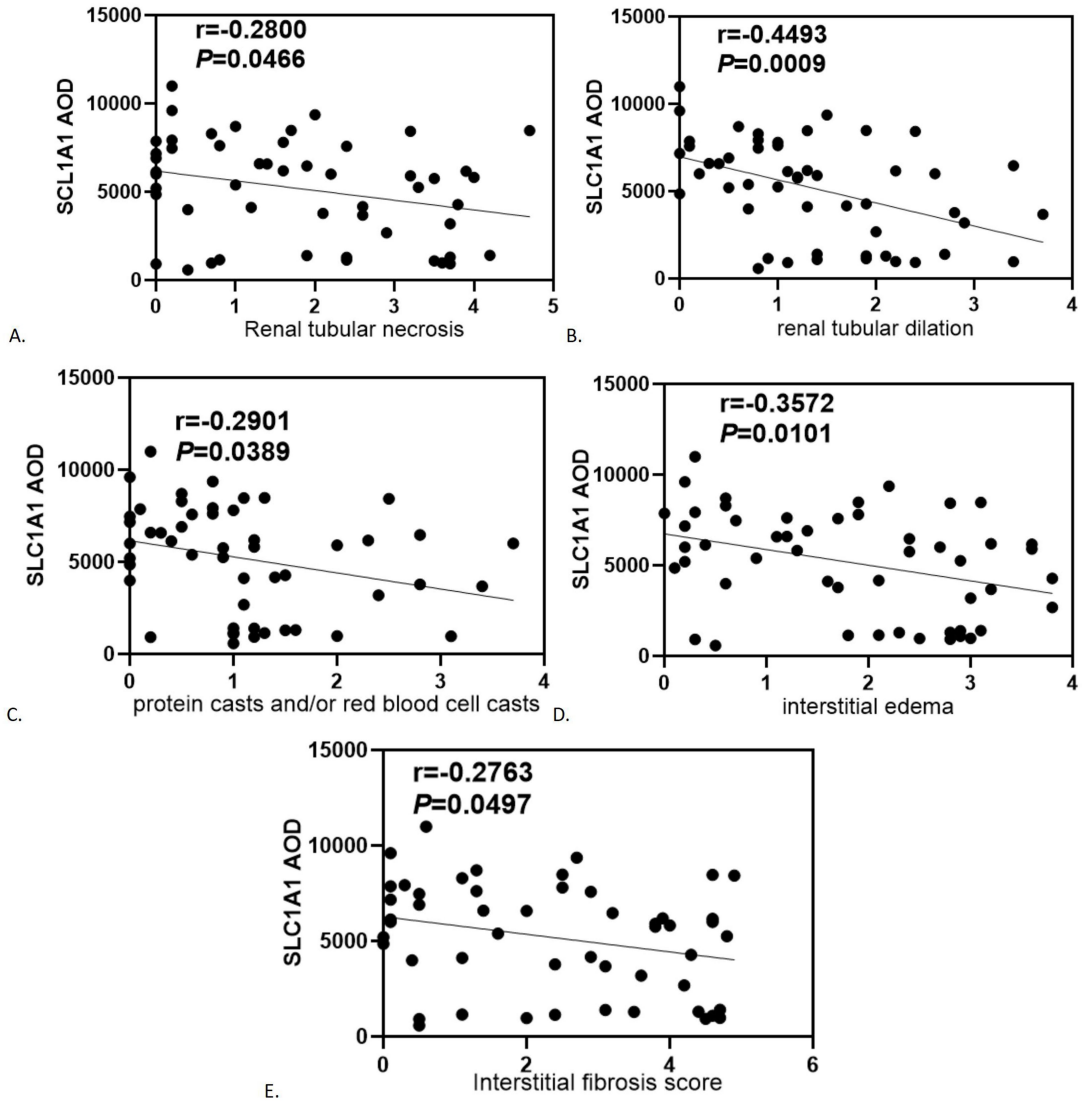
Correlation analysis of statistically significant SLC1A1 with renal tubular-interstitial injury severity score: **(A)** Renal tubular necrosis; **(B)** Renal tubular dilation; **(C)** Protein casts and/or red blood cell casts; **(D)** Interstitial edema; **(E)** Interstitial fibrosis score.

SLC1A1 was detected in both healthy and RIF tissues, primarily localized in the proximal tubules, particularly at the brush borders (see Figure 2A). Some presence was also noted in distal tubules and collecting ducts, predominantly at the membrane level (see Figure 2B). In instances of advanced fibrosis, SLC1A1 was observed in certain interstitial lymphocytes (refer to Figure 2C), along with nonspecific staining in various tubular secretions. Images analysis revealed that the AOD values of SLC1A1 were significantly lower in the RIF cohort compared to healthy controls (*t* = 2.260, *p* = 0.0283; see Figure 2D), with a notable decrease observed in the severely injury subgroup (*t* = 2.794, *p* = 0.0095; see Figure 2G). The mild and moderate injury groups exhibited decreasing trends, although these were not statistically significant (*p* > 0.05; see Figures 2E,F). Correlation assessments revealed negative relationships between SLC1A1 levels and scores for tubular necrosis, dilation, casts, interstitial edema, and fibrosis (*p* < 0.05, see Figures 3A–E). Among these factors, only renal tubular dilation exhibited a moderately correlation, while the others showed weak correlation. The degree of vacuolar degeneration, atrophy and inflammatory cell infiltration were not significantly correlated with SLC1A1 expression (*p* > 0.05).

### 3.3 SLC1A1 expression in cell models

**Figure 4:**
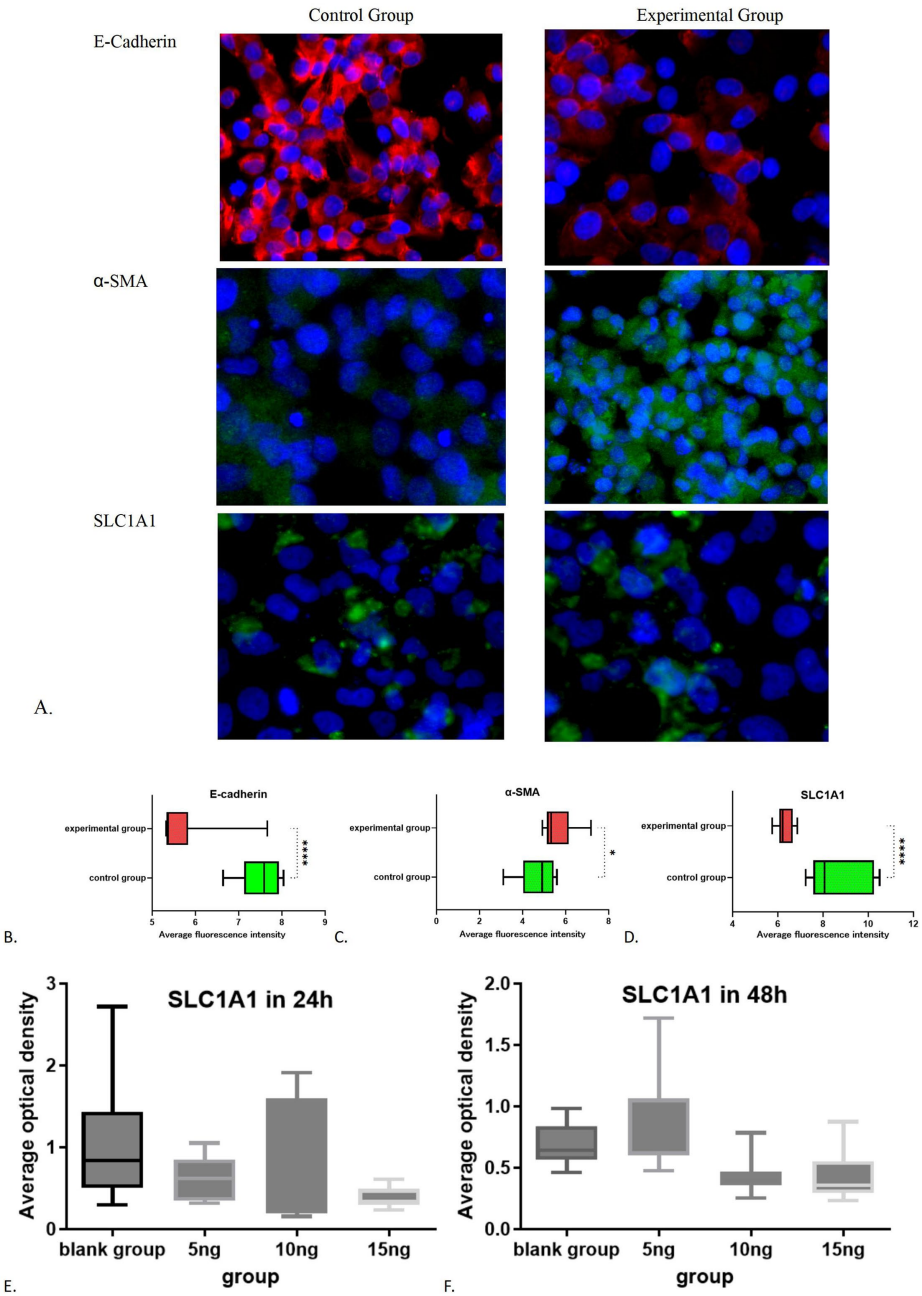
Immunofluorescence results of the control group and experimental group: **(A)** Immunofluorescence images of experimental group and control group; **(B)** Comparative statistical results of E-cadherin between the experimental group and the control group; **(C)** Comparative statistical results of *α*-SMA between the experimental group and the control group; **(D)** Comparative statistical results of SLC1A1 between the experimental group and the control group; **(E)** SLC1A1 in 24 h; **(F)** SLC1A1 in 48 h. *p < 0.05; ****p < 0.0001.

Both the experimental and control cell groups exhibited expression of E-cadherin, *α*-SMA, and SLC1A1 (refer to Figure 4A). The average optical density (AOD) of E-Cadherin expression in the blank group was measured at 7.4861 ± 0.4692, whereas the AOD value in the experimental group was recorded at 5.7124 ± 0.7579. The difference between the two groups was statistically significant (*t* = 6.292, 95% confidence interval was −2.366 to −1.181, *p* < 0.0001). Additionally, the AOD value of *α*-SMA in the blank group was 4.6955 ± 0.8573, while in the experimental group, it was 5.6495 ± 0.7881. The difference was also statistically significant (*t* = 2.591, 95% confidence interval: 0.1803 to 1.728, *p* = 0.0185). Notably, the fluorescence intensity of E-cadherin in the experimental group significantly decreased (Figure 4B), while the level of *α*-SMA increased (Figure 4C), indicating that epithelial mesenchymal transition (EMT) was effectively induced, thus was successfully establishing the fibrosis model. Furthermore, the AOD value of SLC1A1 expression in the blank group was 8.6404 ± 1.2755, compared to 6.3068 ± 0.3486 in the experimental group. SLC1A1 levels were significantly lower in the experimental cells (Figure 4D), with the difference between the two groups being statistically significant (*t =* 5.581, 95% confidence interval: −3.212 to −1.455, *p* < 0.0001). Multivariate analysis of variance showed that a significant difference (*p* < 0.05) in the effect of varying doses of TGF-β1 stimulation on SLC1A1 in renal tubular epithelial cells. Additionally, a significant decrease observed after 24 h of treatment with 15 ng/mL TGF-β1 and after 48 h of treatment with 10 ng/mL and 15 ng/mL TGF-β1 (*p* < 0.05). Treatments with 5 ng/mL TGF-β1 after 24 and 48 h and 10 ng/mL TGF-β1 after 24 h did not show significant reductions (*p* > 0.05, Figures 4E,F). The effects of dissimilar concentrations of TGF-β1 on SLC1A1 expression are shown in Table 2.

**Table 2:** The effects of dissimilar concentrations of TGF-β1 on SLC1A1 expression.

Groups (various TGF-β1 treated)		24 h-AOD	48 h-AOD	Multivariate analysis of variance	Univariate analysis	
					*t*-value (24 h vs. 48 h)	*p*-value (24 h vs. 48 h)
Blank group	0 ng/ml	1.0610	0.6811	Dose effect: *F* = 3.923 *p* = 0.0129 Time effect: 24 h (*F* = 2.736 *p* = 0.0577) 48 h (*F* = 5.996 *p* = 0.0020)	1.607	0.1255
Experimental group	5 ng/ml	0.6313	0.8271		1.266	0.2217
	10 ng/ml	0.7401	0.4406**		1.269	0.2206
	15 ng/ml	0.3806**	0.4334**		0.7128	0.4851

** Compare with the blank group at the same time, *p* < 0.01.

### 3.4 Amino acid and ion concentration changes during fibrosis

**Figure 5:**
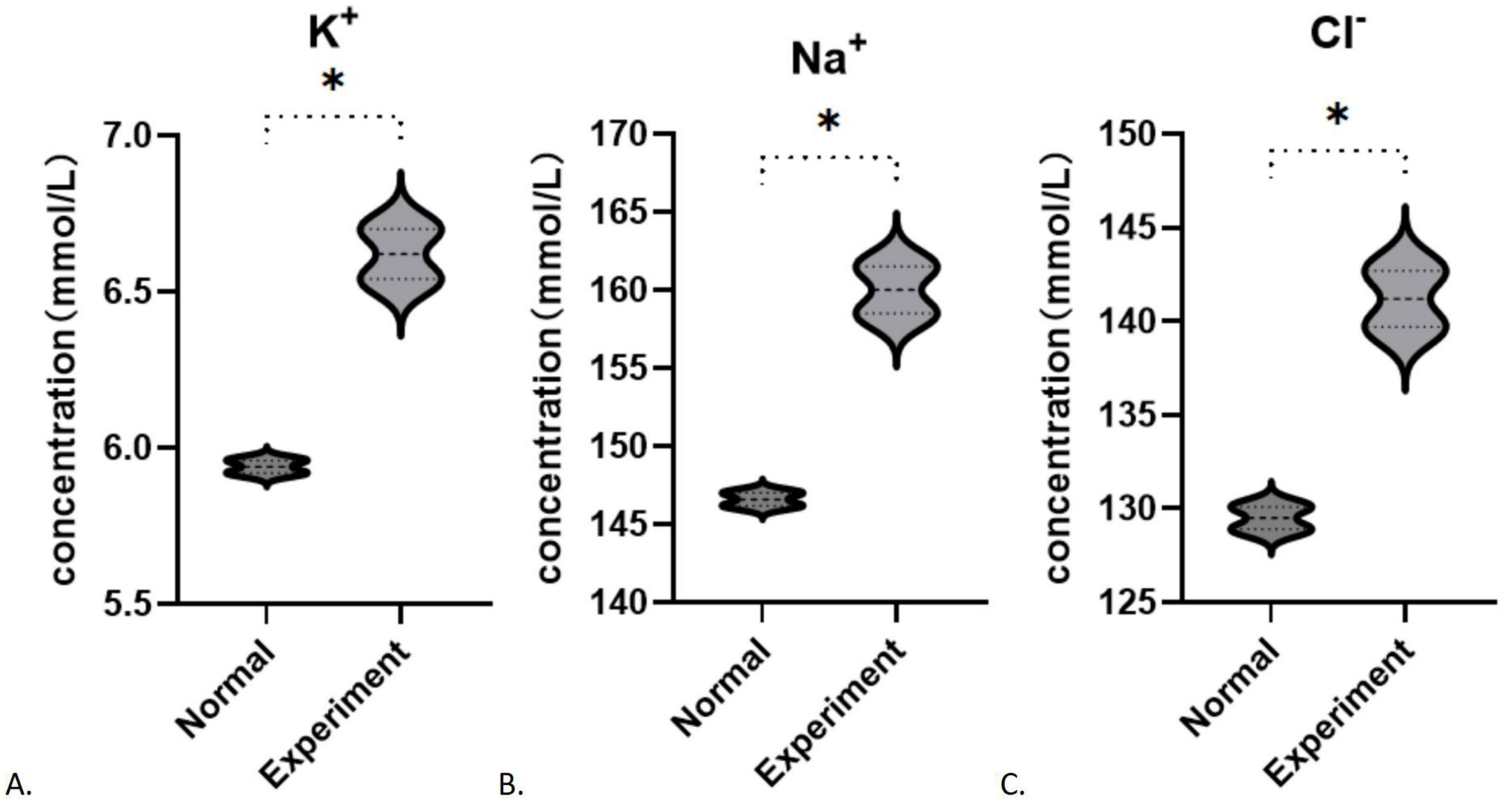
Changes in ion concentration during fibrosis: **(A)** K^+^; **(B)** Na^+^; **(C)** Cl^−^. *p < 0.05.

**Figure 6:**
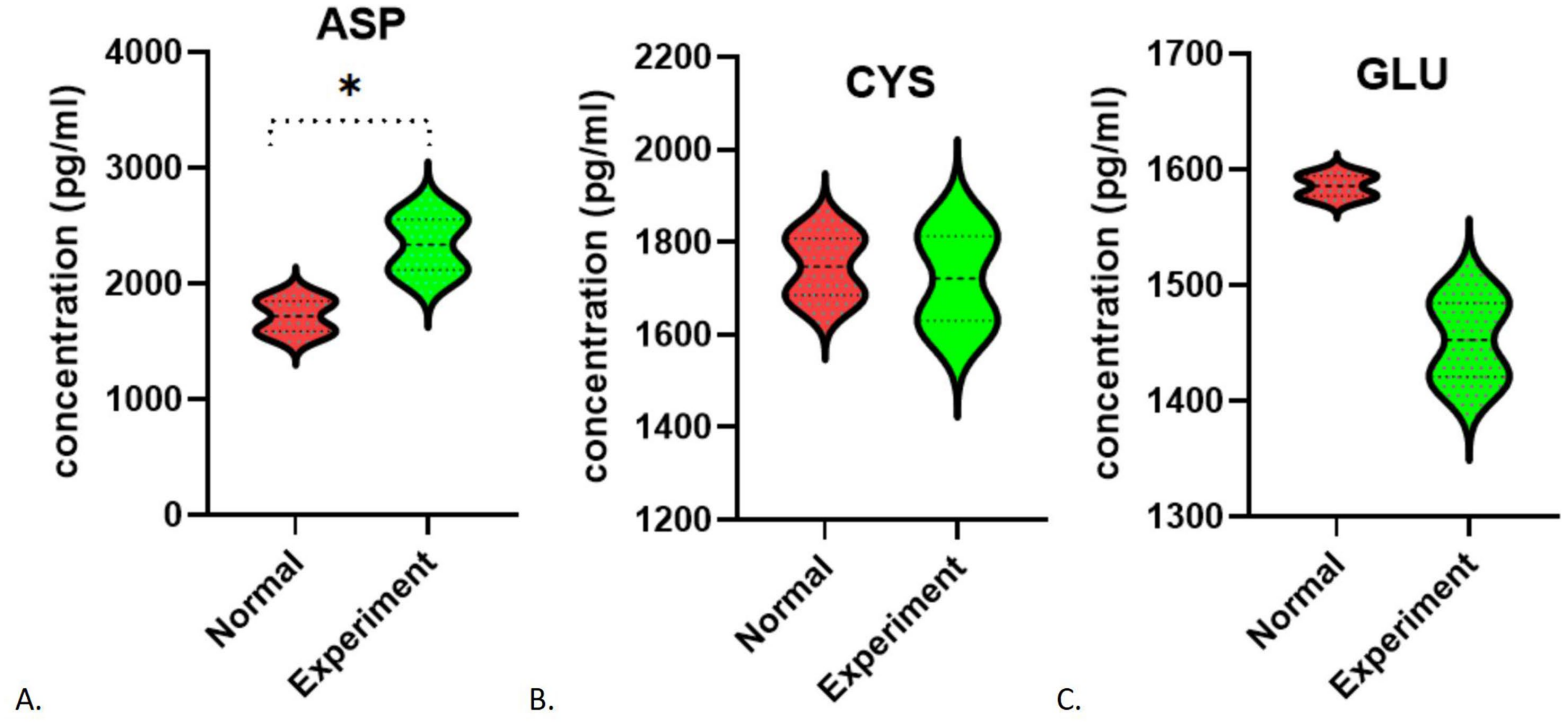
Changes in amino acid concentration during fibrosis (including standard curves): **(A)** Aspartic acid; **(B)** Cysteine; **(C)** Glutamate. *p < 0.05.

After 48 h of cultivation, the potassium ion concentration in the control group was measured at 5.940 ± 0.028 (mmol/L), while the experimental group exhibited a concentration of 6.620 ± 0.113 (mmol/L). The difference between the two groups was statistically significant (*t* = 8.246, *p* = 0.0144). Similarly, the sodium ion concentration in the control group was 146.6 ± 0.566 (mmol/L), whereas the experimental group showed a concentration of 160 ± 2.121 (mmol/L). with a statistically significant difference (*t* = 8.632, *p* = 0.0132). The chloride ion concentration in the control group was recorded at 129.5 ± 0.849 (mmol/L), while in the experimental group had a concentration of 141.2 ± 2.121 (mmol/L), which also demonstrated a statistically significant difference (*t* = 7.242, *p* = 0.0185). In contrast to the control group, the experimental subjects displayed a minor increase in potassium levels (*p* < 0.05) alongside a notable increase in sodium and chloride concentrations (*p* < 0.05; Figures 5A–C). After 48 h of cultivation, the concentration of aspartic acid in the control group was 1717.75 ± 184.20 (pg/mL), while the experimental group exhibited a concentration of 2335.86 ± 309.00 (pg/mL), with a statistically significant difference (*t* = 0.3.436, *p* = 0.0139). The concentration of cysteine in the control group was 1746.86 ± 86.83 (pg/mL), whereas the experimental group showed a concentration of 1721.6 ± 129.3 (pg/mL), with no statistically significant difference between the groups (*t* = 0.3244, *p* = 0.7567). The concentration of glutamate in the control group was 1585.73 ± 12.20 (pg/mL), while in the experimental group it was 1452.72 ± 45.08 (pg/mL), indicating no statistically significant difference (*t* = 4.028, *p* = 0.0565). Analysis of amino acids revealed an increase in aspartic acid (*p* < 0.05, Figure 6A), stable levels of cysteine (*p* > 0.05, Figure 6B), and a slight decrease in glutamic acid within the experimental cells (*p* > 0.05, Figure 6C).

### 3.5 SLC1A1-mediated glutamate uptake during fibrosis

**Figure 7:**
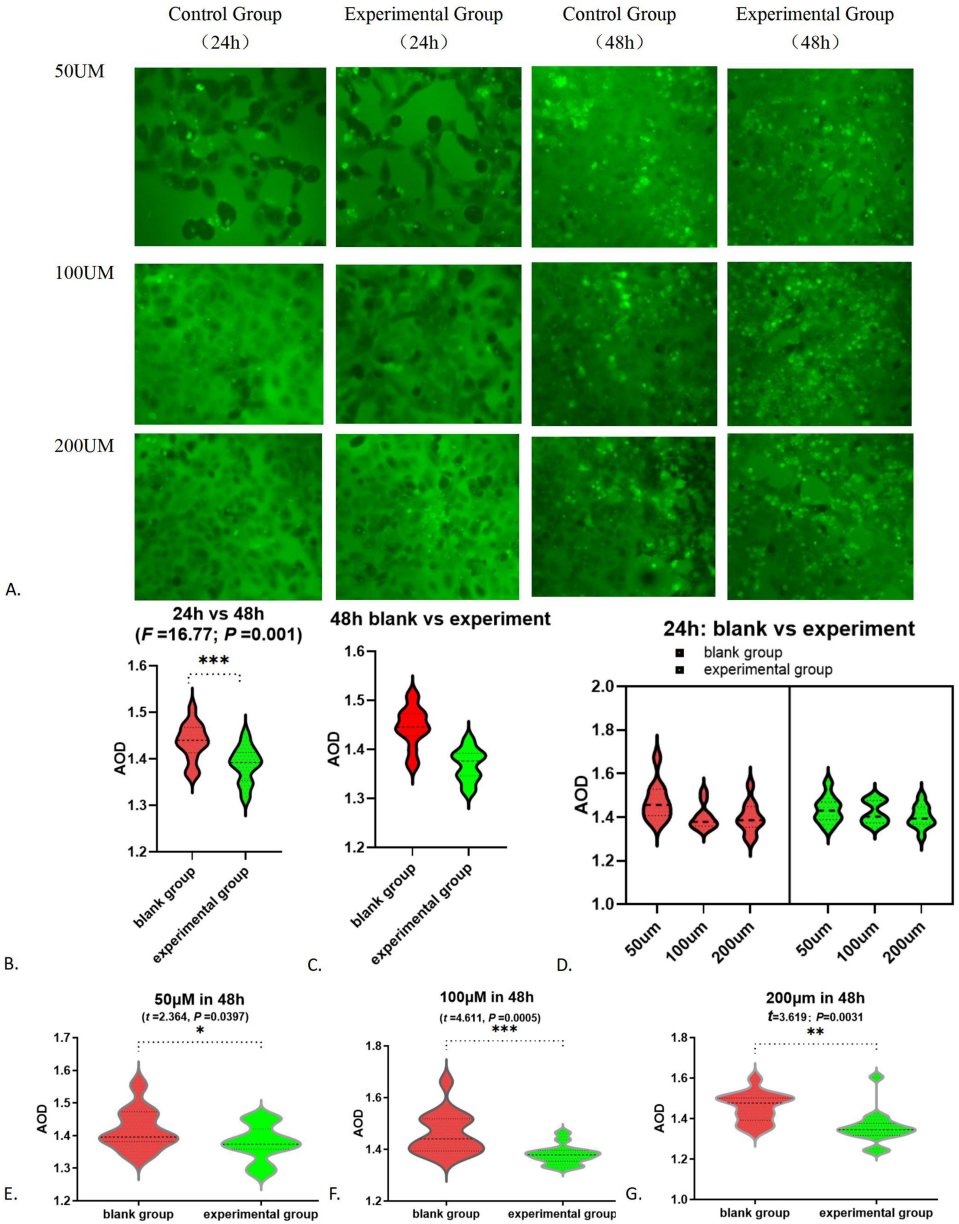
Alteration in glutamate absorption function: **(A)** FITC-glutamic acid absorption fluorescence chart; **(B)** FITC-glutamate absorption fluorescence AOD results-24 h vs. 48 h; **(C)** Comparison of glutamate absorption between the white group and the experimental group at 48 h; **(D)** FITC-glutamate absorption in 24 h; **(E)** 50 μM in 48 h; **(F)** 100 μM in 48 h; **(G)** 200 μM in 48 h. *p < 0.05; **p < 0.01; ***p < 0.001.

Following the addition of 0 and 25 μM FITC-glutamate, minimal fluorescence was detected. In contrast, cells treated with 50, 100, and 200 μM FITC-glutamate exhibited notable fluorescence labeling. After 24 h, most of the FITC-glutamate was found between the cells and was dispersed; by 48 h, a substantial quantity had penetrated the cells and was found in droplet formations, leading to a marked reduction of fluorescent dyes in the intercellular spaces (Figure 7A; Table 3). Statistically significant differences were noted between the 24 h and 48 h observations (*F* = 16.77, *p* = 0.001, Figure 7B). At 48 h, the absorption of glutamate in the experimental group was significantly lower than that in the control group (*F* = 34.33, *p* < 0.0001, Figure 7C). In contrast, no significant difference was observed between the groups at 24 h (*F* = 0.0418, *p* = 0.8400). Multivariate analysis indicated that the dose–response effect was statistically significant only at 50 μM (*F* = 5.137 *p* = 0.0347), whereas no significant difference were found between the 100 μM and 200 μM dose (*p* > 0.05). The results at the 24 h mark showed no significant variation between the control group and those treated with FITC-glutamate at concentrations of 50, 100, and 200 μM (*p* > 0.05) (Figure 7D). At the 48 h time point, the differences between the experimental and control groups were particularly pronounced at the 50, 100, and 200 μM concentrations (Figures 7E–G).

**Table 3:** Study on the uptake of glutamate mediated by SLC1A1 during fibrosis process.

Groups		24 h-AOD	48 h-AOD	Multivariate analysis of variance	Univariate analysis	
					*t*-value (24 h vs. 48 h)	*P*-value (24 h vs. 48 h)
Blank group	50 μM	1.30 ± 0.54	1.42 ± 0.06	Dose effect: 50 μM (*F* = 5.137 *p* = 0.0347) 100 μM (*F* = 0.9201 *p* = 0.3444) 200 μM (*F* = 1.164 *p* = 0.2942) Time effect: 24 h (*F* = 0.0418 *p* = 0.8400) 48 h (*F =* 34.33 *p* < 0.0001)	2.096	0.0478
	100 μM	1.19 ± 0.50	1.46 ± 0.08		6.387	<0.0001
	200 μM	1.21 ± 0.51	1.46 ± 0.07		5.557	<0.0001
Experimental group	50 μM	1.26 ± 0.53	1.38 ± 0.38*		8.552	<0.0001
	100 μM	1.22 ± 0.51	1.38 ± 0.04***		6.274	<0.0001
	200 μM	1.22 ± 0.51	1.36 ± 0.08**		7.475	<0.0001

*** Compare with the blank group at the same time: * *p* < 0.05; ** *p* < 0.01; *** *p* < 0.001.

## 4 Discussion

This research integrates both clinical and laboratory experiments to investigate changes in SLC1A1 during the development of renal interstitial fibrosis and their impact on amino acid metabolism and ion levels. Findings from cell culture experiments demonstrate a decline in SLC1A1 during fibrosis, with its expression inversely related to the severity of renal fibrosis. This trend may stem from various interacting mechanisms, including the activation of TGF-β1 signaling, metabolic and ion homeostasis imbalances, oxidative stress, and potential epigenetic influences (34–38). Histological analyses revealed that SLC1A1 protein levels are inversely associated with renal tubular necrosis, dilation, the presence of protein and/or red blood cell casts, interstitial edema, and fibrosis scores, but show no correlation with vacuolar degeneration of renal tubular epithelial cells, tubular atrophy, or interstitial cell infiltration. These findings suggest that TGF-β1 modifies SLC1A1 expression by altering the characteristics of renal tubular epithelial cells during fibrosis. Additionally, significant damage or necrosis of these cells may further contribute to the reduction of SLC1A1, impairing its normal function. Interestingly, no significant difference in SLC1A1 expression was observed between moderate and severe injury groups, possibly due to SLC1A1 expression in lymphocytes within the renal interstitium during severe injury, which could influence the overall expression profile. Future targeted analyses of renal tubular epithelial cells will be performed to eliminate this possibility. Furthermore, it suggests that in cases of moderate to severe fibrosis, stromal cells, particularly well-studied macrophages (39–41), may also influence outcomes through SLC1A1-expressed proteins. Additional experiments are necessary to elucidate further mechanisms and specific pathways. Impaired renal tubular reabsorption and secretion is a key factor in the pathogenesis of chronic kidney disease. The diminished expression of SLC1A1, a critical transport protein, in renal tubular epithelial cells adversely impacts renal tubular function, particularly in the proximal tubules, potentially leading to amino acid loss in urine and proximal tubular acidosis, which contribute to systemic environmental disturbances. By preserving more renal tubular cells and restoring the function of transport proteins like SLC1A1, the progression of these disturbances can be mitigated. SLC1A1 represents a significant target for preserving of renal function.

To delve deeper into the role of the SLC1A1 protein, which functions as a transporter for amino acids and ions, we analyzed the levels of specific amino acids (such as glutamic acid, cysteine, and aspartic acid) and ions (including potassium, chloride, and sodium) present in the cell culture medium during the fibrosis process. Our findings indicated notable changes in the concentrations of both amino acids and ions. In the context of ion concentration variations, the TGF-β1-induced fibrosis study suggested that TGF-β1 could induce an acid–base imbalance by influencing transporters such as SLC1A1 and activating specific signaling pathways (42), resulting in a minor rise in extracellular K^+^ levels and significant increases in Na^+^ and Cl^−^ levels. A reduction in SLC1A1 protein correlates with diminished internalization of sodium and hydrogen ions, which consequently raises their extracellular concentrations, aligning with our observations. Nevertheless, the decreased expression of SLC1A1 should also lead to a reduced capacity for potassium ion excretion, implying a lower extracellular potassium concentration. Contrary to this expectation, our results showed an increase in extracellular potassium levels. This suggests that, in addition to SLC1A1’s influence, other ion transport proteins and channels (such as SLC12A4, ATPase Na+/K + transporting β1) are also involved (43). Moving forward, we aim to explore this phenomenon further by utilizing SLC1A1 inhibitors and examining additional ion transporters or channels.

In terms of changes in amino acid levels, the experimental group showed a rise in aspartic acid, stable levels of cysteine, and a decline in glutamic acid. Since the primary role of the SLC1A1 protein is to facilitate the transport of extracellular glutamate into cells, a reduction in this protein would typically result in higher extracellular glutamate levels. However, our findings contradict this expectation. We did not detect an increase in glutamate concentration, which could be attributed to factors such as a small sample size and a brief observation period. Other amino acid transporters (SLC16A10, SLC3A2, SLC7A7, and so on) may also be involved in this outcome (44). We noted an elevation in extracellular aspartic acid concentration, while cysteine levels remained largely unchanged. This suggests that, under these conditions, SLC1A1 can still influence aspartic acid levels, but its impact on cysteine is minimal. Nevertheless, due to the limited scope of our study, further research is necessary to confirm the generalizability of these findings.

An analysis of the findings regarding ion and amino acid concentrations reveals that the SLC1A1 protein diminishes during the fibrosis process. Notably, fluctuations in ion levels are more readily apparent than those in amino acid concentrations. In the initial phases of fibrosis, SLC1A1 plays a crucial role by modulating ion and acid–base equilibrium, thereby influencing intracellular ion levels. In subsequent stages, we will focus on a detailed investigation of iron and copper ions, which are currently under significant scrutiny, along with the cell death mechanisms associated with these ions (45).

Research on glutamate transport capacity revealed that, at the initial 24 h mark, fluorescently tagged glutamate primarily accumulated in the intercellular spaces, whereas after 48 h, a greater amount was found within the cells. The transport of glutamate through SLC1A1 was shown to be concentration-dependent, with higher levels of glutamate leading to increased transport capacity. These findings align closely with the known role of SLC1A1. At a concentration of 50 μM, the effects are evident; however, at concentrations of 100 μM and 200 μM, the dosage appears to have no significant impact. This observation suggests that there is a finite number of SLC1A1 transporters and a limit to the transport of amino acids. When the concentration of amino acids reaches a certain threshold, the transport capacity cannot be further increased. In the context of fibrosis, a decrease in SLC1A1 expression occurs, negatively impacting its transport capabilities. As a result, both the efficiency of glutamate transport and the average optical density decline align with our predictions.

To summarize, this research suggests that SLC1A1 expression is reduced in the fibrosis group, whether in tissue samples or cultured cells, and this reduction is inversely correlated with the progression of renal interstitial fibrosis. The downregulation of SLC1A1 is influenced by various factors, including TGF-β1. Alterations in SLC1A1 levels result in modifications to amino acid transport and ion levels. Additionally, compensatory mechanisms, such as increased passive diffusion and the involvement of alternative transporters, contribute to this phenomenon, helping to clarify the seemingly paradoxical relationship between the decreased SLC1A1 expression and the heightened transport capacity observed in certain situations. These results underscore the potential of SLC1A1 as a promising target for therapeutic interventions aimed at enhancing kidney function.

## 5 Shortcomings and research prospects

While this research has yielded some findings, it also has notable limitations due to constraints in time and resources: ① The investigation was confined to 51 samples from two hospitals located in Sichuan and Shandong, which restricts the diversity related to ethnicity and geographic factors. Future research should incorporate a broader range of regions and medical facilities to enhance the variety and number of samples, thereby improving the generalizability of the findings. ② The TGF-*β*1-induced HK-2 cell model used to mimic renal interstitial fibrosis is a well-established model that effectively represents the epithelial-mesenchymal transition (EMT) and the activation of myofibroblasts. However, further validation is needed to determine if this model accurately reflects the role of SLC1A1 in renal interstitial fibrosis. Future studies will involve specific knockout or overexpression experiments of SLC1A1 to directly assess its involvement in the fibrosis process. Upcoming research may explore the following areas: ① Analysis of molecular mechanisms: Investigating whether SLC1A1 influences fibrosis by modulating GSH synthesis or the TGF-β/Smad signaling pathway. ② Potential for clinical application: Creating a kidney-targeted nanoparticle delivery system based on SLC1A1, in conjunction with SGLT-2 inhibitors, to enhance anti-fibrotic effects while minimizing the risk of toxicity associated with high-dose interventions.

## Data Availability

The original contributions presented in the study are included in the article/supplementary material, further inquiries can be directed to the corresponding author.

## Author’s note

Fibrotic renal tissues and TGF-β1-induced renal tubular epithelial cells were utilized to assess the extent and progression of fibrosis. Our findings indicate that SLC1A1 expression was reduced in both the affected tissues and cells. The downregulation of SLC1A1 in renal interstitial fibrosis shows an inverse relationship with fibrosis severity and affects both glutamate uptake and the balance of extracellular ions, suggesting its potential as a therapeutic target to improve kidney function.
